# Artificial Intelligence as a Consent Aid for Carpal Tunnel Release

**DOI:** 10.7759/cureus.63041

**Published:** 2024-06-24

**Authors:** James Brock, Richard Roberts, Matthew Horner, Preetham Kodumuri

**Affiliations:** 1 Trauma and Orthopedics, Wales Deanery, Cardiff, GBR; 2 Trauma and Orthopedics, Wrexham Maelor Hospital, Wrexham, GBR; 3 Trauma and Orthopedics, Robert Jones and Agnes Hunt Hospital, Oswestry, GBR

**Keywords:** chatgpt, carpal tunnel release, consent, large language models, artificial intelligence

## Abstract

Background

Hand surgeons have been charged with the use of diverse modalities to enhance the consenting process following the Montgomery ruling. Artificial Intelligence language models have been suggested as patient education tools that may aid consent.

Methods

We compared the quality and readability of the Every Informed Decision Online (EIDO) patient information leaflet for carpal tunnel release with the artificial intelligence language model Chat Generative Pretrained Transformer (GPT).

Results

The quality of information by ChatGPT was significantly higher using the DISCERN score, 71/80 for ChatGPT compared to 62/80 for EIDO (p=0.014). DISCERN interrater observer reliability was high (0.65) using the kappa statistic. Flesch-Kincaid readability scoring was 12.3 for ChatGPT and 7.5 for EIDO, suggesting a more complex reading age for the ChatGPT information.

Conclusion

The artificial intelligence language model ChatGPT produces high-quality information at the expense of readability when compared to EIDO information leaflets for carpal tunnel release consent.

## Introduction

Enhanced consent in hand surgery refers to the use of diverse modalities to aid consultation and paperwork to obtain informed consent [1]. In carpal tunnel release surgery, median nerve injury is the greatest source of litigation, and the associated lack of consent is felt to contribute toward a negligence claim [2].

Since the Montgomery ruling in 2015, surgeons have been charged with explaining all treatment options and identifying risks that are subjectively most important to patients [3]. Audio-visual aids such as patient information leaflets, eLearning videos, and structured consent forms have been shown to improve patient immediate recall and therefore facilitate higher-level conversation surrounding material risk [4].

In carpal tunnel release surgery, written information increases risk recall rates, with a written consent form shown to influence most patients' final decision [5,6]. Every Informed Decision Online (EIDO) leaflets are often viewed as a medicolegal gold standard of written information provision by consultant surgeons; they have been shown to increase the patient's understanding of risks and satisfaction during the consent process [7].

Artificial intelligence research in hand surgery has boomed in recent years with potential applications highlighted in fracture detection and automated screening [8]. Chat Generative Pretrained Transformer (GPT) is a natural language processing. Artificial intelligence technology has been suggested as a patient education tool by providing information equivalent to common web searches [9].

This paper aims to look at the role of artificial intelligence as a consent aid in carpal tunnel surgery and specifically to assess whether the quality and readability of the information generated by ChatGPT is comparable to that of a carpal tunnel-specific EIDO leaflet.

## Materials and methods

Our null hypothesis was that there is no difference in the quality and readability of information provided by EIDO leaflets and ChatGPT when explaining carpal tunnel release.

The EIDO carpal tunnel release leaflet (OS05 Lite, expiring January 2024) was taken as the consenting standard. The 10 specific question domains set out in the EIDO leaflet were used as input prompts for the artificial intelligence model: ChatGPT 3.5. The responses from ChatGPT were then amalgamated into a single document. Both documents were then blinded by formatting each without identifying information in identical layout and font.

The quality of information was assessed using the DISCERN scoring system in a blinded manner. The DISCERN score was developed by Charnock et al. and uses 16 domains to assess relevance, bias, completeness, and quality of information within a document [10]. Scores range from 1 to 5 per domain, with a maximum score of 80 indicating the high quality of information. The blinded documents were then presented to five healthcare clinicians (two consultant hand surgeons, two orthopedic specialist registrars, and one senior house officer) for qualitative analysis.

The readability of information was assessed using the Flesch-Kincaid grading and Gunning fog index [11,12]. Both systems use an average number of syllables per word and an average number of words per sentence to determine the reading age of the text. A Flesch-Kincaid score is generated out of 18 with a corresponding reading age: the lower the score the easier the readability. The Gunning fog index is interpreted out of 17 in the same fashion. Flesch-Kincaid also generates a reading ease score out of 100, which is generated afterward, with 100 being easily readable. Both calculators provide information about the number of syllables and words used in the calculation.

Statistical analysis was performed using the information quality DISCERN scores, to look for significant differences between information sources and rater agreements. A paired Student's t-test was used to compare domain scores between ChatGPT and the EIDO leaflet, with a p-value of <0.05 taken as statistically significant. Interobserver reliability was calculated using the kappa statistic [13]. Kappa scores range from -1 to +1. Perfect agreement is 0.81 to 1; no agreement is a score of <0.

## Results

Quality of information

The mean DISCERN score was 71/80 for ChatGPT and 62/80 for EIDO. A DISCERN score of >63 indicates excellent quality, with 51-62 categorized as good. The mean score per domain was 4.4/5 for ChatGPT and 3.9/5 for EIDO. A score of 5 agrees with the statement, whereas 1 disagrees. The mean scores for overall quality were 4.8 for ChatGPT and 4.2 for EIDO. An overall score of 5 is high quality, with 1 being low quality. The lowest scores were seen in the two questions assessing where information was sourced from and when information was obtained, with equally poor performance from both data sets. The mean DISCERN scores per domain for ChatGPT and the EIDO leaflet are summarized in Table 1.

**Table 1:** Summary of Mean DISCERN Score per Domain DISCERN scoring per question for ChatGPT and EIDO quality of information (1, no; 3, somewhat; 5, yes) (overall 1, low quality; 5, high quality) GPT, Generative Pretrained Transformer; EIDO, Every Informed Decision Online

	ChatGPT	EIDO
Are the aims clear?	5	3.8
Does it achieve aims?	4.8	4.2
Is the information relevant?	5	4.4
Clear sources of information?	3	2.4
Clear when information was reported?	3	2.2
Balanced and unbiased?	4.8	4.4
Details of additional sources of information?	3.6	3.2
Refer to areas of uncertainty?	4.8	3.4
Describe each treatment?	5	3.8
Benefits of each treatment?	4.2	3.6
Risks of each treatment?	4.4	4.2
Describe no treatment?	4.8	4.4
Describes the quality-of-life effect of the treatment?	4	4
Clear there are more than one treatment options?	5	4.8
Does it provide support for shared decision-making?	4.8	4.6
Overall quality?	4.8	4.2

Readability of information

The total word count for each set of information was 1843 for ChatGPT and 611 for EIDO. The mean number of words per sentence was 14.1 for ChatGPT and 10.9 for EIDO, with 1.9 syllables per word for ChatGPT and 1.6 for EIDO.

The readability of the information provided by the EIDO leaflet was easier than that of ChatGPT. The Flesch-Kincaid score was 12.3 for ChatGPT and 7.5 for EIDO. Further, the reading ease score was 31.8/100 for the ChatGPT information and 60.4/100 for the EIDO leaflet. The Gunning fog index was 14.22 for ChatGPT and 11.02 for EIDO. Overall reading age was classified as university level for ChatGPT and year 9/10 for EIDO. The readability of information scores is summarized in Table 2.

**Table 2:** Summary of Information Readability Summary of readability scores using the Flesch-Kincaid grading and Gunning fog index (Flesch-Kincaid score, out of 18; reading ease score, out of 100; Gunning fog index, out of 17) GPT, Generative Pretrained Transformer; EIDO, Every Informed Decision Online

	ChatGPT	EIDO
Kincaid score	12.3	7.5
Reading ease score	31.8	60.4
Reading level	University	Year 9/10
Words per sentence	14.1	10.9
Syllables per word	1.9	1.6
Gunning fog index	14.22	11.02
>3-syllable words	443	75
Word count	1843	611

Statistical analysis

Student's t-test for the mean scores per domain was 0.014, implying a statistically significant difference in the average DISCERN quality of information score. The interobserver reliability kappa score was 0.65 overall for both leaflets, which may be interpreted as substantial agreement between raters. The interrater coefficient was 0.725 for the ChatGPT sample and 0.575 for the EIDO leaflet.

## Discussion

The role of artificial intelligence in hand surgery is expanding; the most promising and functional areas seem to be patient education and as radiological adjuncts [14]. Consent is a multistage process, where repeated patient involvement facilitates higher-level discussion around material risk. Reading age-appropriate eLearning videos appears to be a superior audio-visual aid to enhance patient satisfaction with the consenting process [15]. The augmentation of this electronic process with artificial intelligence tools such as ChatGPT could be used to aid consent in carpal tunnel release surgery, by providing information to patients and answering specific questions in an interactive manner.

The overall quality of information provided by both ChatGPT and the EIDO leaflet was high. The quality of answers provided by ChatGPT was felt to be better by all clinicians using the DISCERN grading system. Anecdotal comments from assessors were that the responses generated by ChatGPT were more detailed and descriptive of alternative interventions and complications. Both provided balanced and unbiased information for carpal tunnel surgery and directed the patient to discuss further with their clinician. The overall reading age for both ChatGPT and the EIDO leaflet was well above the national average for the United Kingdom of nine years old. This reflects previous research on consent forms in hand surgery [16]. The information provided in the EIDO leaflet was more concise than ChatGPT and at a lower reading age.

Limitations were present in both sources of information. While both sources alluded to persistent symptoms or pain, neither specifically mentioned complex regional pain syndrome. Further, while mentioning potential symptoms of nerve damage, the EIDO leaflet did not explicitly mention this term. Another criticism of both sources was suggesting that the benefit of surgery would definitely resolve numbness and weakness, a patient expectation that is often not the case and should be addressed preoperatively [17]. Finally, although being more concise and readable, the EIDO leaflet only suggested two conservative treatment options compared to five by ChatGPT, suggesting that the EIDO information may not be adequate when considering the Montgomery requirements. Further, our study could have been improved by utilizing the recent ChatGPT 4.0 model and modifying inputs to affect reading age.

We suggest that an EIDO information leaflet and ChatGPT, however, can be used in conjunction with each other. EIDO can be used to provide concise readable information to the patient, while ChatGPT could be used following this to facilitate more detailed and tailored information in a conversational manner. Utilizing both consent aids could thus facilitate better subsequent consultation around material risk with a clinician.

## Conclusions

The artificial intelligence language model ChatGPT produces high-quality information at the expense of readability when compared to EIDO information leaflets for carpal tunnel release. Future research should look at modifying inputs to improve artificial intelligence responses and assess this in a patient setting.
